# Supplementary material for: Optimization of Mapping Tools and Investigation of Ribosomal RNA Influence for Data-Driven Gene Expression Analysis in Complex Microbiomes
Source: Microorganisms. 2025 Apr 26;13(5):995. doi: 10.3390/microorganisms13050995 (PMC12113988; doi:10.3390/microorganisms13050995)
Supplement: Supplementary file 1 [file microorganisms-13-00995-s001.zip › microorganisms-3580823-supplementary.pdf]

Table S1. NGS reads of soil microbiome used in this study.

SRR	sample type	reference
SRR20140048	metagenomic	[13]
SRR20140049	metagenomic	[13]
SRR20140050	metagenomic	[13]
SRR20140051	metagenomic	[13]
SRR20140052	metagenomic	[13]
SRR20140053	metagenomic	[13]
SRR20140054	metagenomic	[13]
SRR20140055	metagenomic	[13]
SRR20140138	metatranscriptomic	[13]
SRR20140139	metatranscriptomic	[13]
SRR20140141	metatranscriptomic	[13]
SRR20140142	metatranscriptomic	[13]
SRR20140143	metatranscriptomic	[13]
SRR20140144	metatranscriptomic	[13]
SRR20140145	metatranscriptomic	[13]
SRR20140146	metatranscriptomic	[13]
SRR22411266	metagenomic	[10]
SRR22411277	metagenomic	[10]
SRR22411293	metagenomic	[10]
SRR22411304	metagenomic	[10]
SRR22411313	metagenomic	[10]
SRR22411314	metagenomic	[10]
SRR22411022	metatranscriptomic	[10]
SRR22411033	metatranscriptomic	[10]
SRR22411020	metatranscriptomic	[10]
SRR22411021	metatranscriptomic	[10]
SRR22411044	metatranscriptomic	[10]
SRR22411045	metatranscriptomic	[10]
SRR22507541	metagenomic	[20]
SRR22507542	metagenomic	[20]
SRR22507543	metagenomic	[20]
SRR22507544	metagenomic	[20]
SRR22506317	metatranscriptomic	[20]
SRR22506319	metatranscriptomic	[20]
SRR22506320	metatranscriptomic	[20]
SRR22506321	metatranscriptomic	[20]
SRR22506322	metatranscriptomic	[20]
SRR22506323	metatranscriptomic	[20]
SRR22506324	metatranscriptomic	[20]
SRR22506325	metatranscriptomic	[20]
SRR22506326	metatranscriptomic	[20]
SRR22506304	metatranscriptomic	[20]

SRR22506327	metatranscriptomic	[20]
SRR22506328	metatranscriptomic	[20]
SRR24888308	metagenomic	[9]
SRR24888310	metagenomic	[9]
SRR24888329	metagenomic	[9]
SRR24888330	metagenomic	[9]
SRR24888635	metagenomic	[9]
SRR24888648	metagenomic	[9]
SRR24887267	metatranscriptomic	[9]
SRR24887221	metatranscriptomic	[9]
SRR24887404	metatranscriptomic	[9]
SRR24887272	metatranscriptomic	[9]
SRR24888495	metatranscriptomic	[9]
SRR24887388	metatranscriptomic	[9]

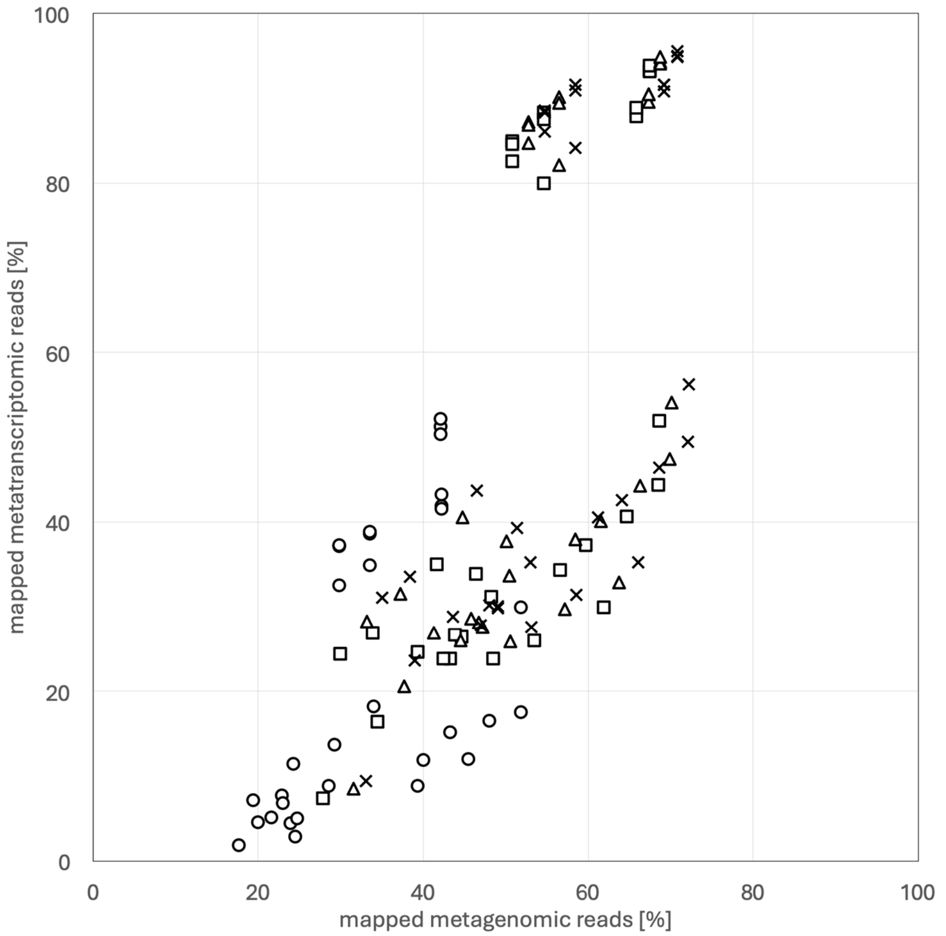

Figure S1. Mapping rates of metagenomic and metatranscriptomic reads using Bowtie2. Setting was --sensitive (circles), --local -L 19 (squares), --very-sensitive-local -L 19 (triangles), or -very-sensitive-local -L 19 -mp 4 (crosses).

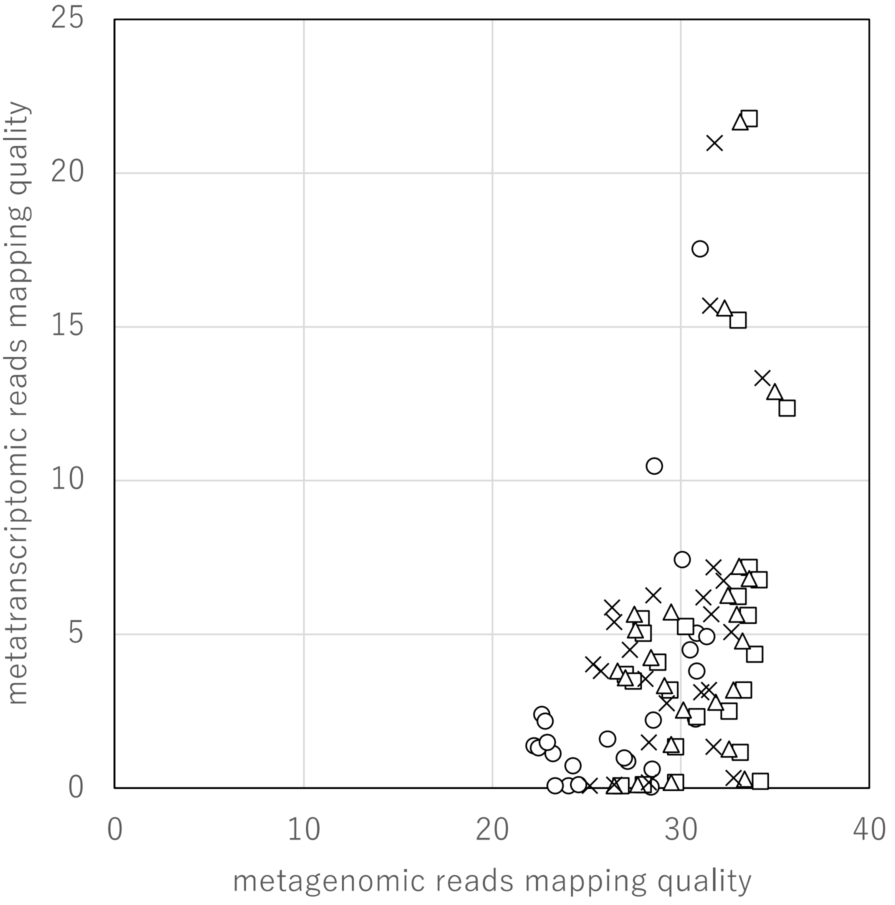

Figure S2. Mapping quality of metagenomic and metatranscriptomic reads using Bowtie2. Setting was --sensitive (circles), --local -L 19 (squares), --very-sensitive-local -L 19 (triangles), or -very-sensitive-local -L 19 -mp 4 (crosses).
